# Supplementary material for: Covalent docking and molecular dynamics simulations reveal the specificity-shifting mutations Ala237Arg and Ala237Lys in TEM beta-lactamase
Source: PLoS Comput Biol. 2022 Jun 27;18(6):e1009944. doi: 10.1371/journal.pcbi.1009944 (PMC9269908; doi:10.1371/journal.pcbi.1009944)
Supplement: S1 Appendix — (PDF) [file pcbi.1009944.s001.pdf]

Appendix S1: Choice of Docking Methodology

To measure the effects of point mutations in TEM's alanine 237 residue on the enzyme's substrate specificity, we elected to utilize Schrodinger's CovDock covalent docking workflow [1]. We defined a beta-lactam opening reaction between the nucleophilic oxygen of serine 70 in TEM and the carbonyl group within the beta-lactam, following previous mechanistic reports [2]. The CovDock methodology we employed is summarized in Fig 1.

As part of the CovDock workflow, we utilized Glide, which is a widely-used docking engine that employs the OPLS3 force-field [3] and a wide variety of scoring functions depending on the desired thoroughness. It uses sequential hierarchical filters to identify possible ligand binding locations within the search space, which is usually the binding site of a receptor whose shape and properties are represented on a grid [1]. Prime is a structure prediction algorithm that uses previously annotated data (when available) and *ab initi*o modeling to predict the conformation of biological macromolecules. Prime's predictive accuracy is greatly increased when modeling small changes to known structures, such as point mutations in proteins [4].

Through covalent docking, we sought to identify poses that are more likely to resemble catalytically-relevant conformations for the docked ligands, as binding modes that do not allow for the formation of a covalent bond with serine 70 are discarded after the initial scoring. Additionally, CovDock allows us to probe potential active site rearrangements in response to the binding of substrates, as structural relaxation steps are conducted after the formation of the covalent bond. This is especially important as TEM's active site is relatively occluded and might shift considerably to allow for the binding of bulkier substrates such as cefixime (Fig S3).

References

1. Introducing CovDock for Covalent Docking | Schrodinger. https://www.schrodinger.com/newsletters/introducing-covdock-covalent-docking.

2. Knox, R.; Lento, C.; Wilson, D. J. Mapping Conformational Dynamics to Individual Steps in the TEM-1 Β-Lactamase Catalytic Mechanism. *Journal of Molecular Biology* **2018,** *430,* 3311–3322.

3. Harder, E. et al. OPLS3: A Force Field Providing Broad Coverage of Drug-like Small Molecules and Proteins. *Journal of Chemical Theory and Computation* **2016,** *12,* 281–296, Publisher: American Chemical Society.

4. Jacobson, M. P.; Pincus, D. L.; Rapp, C. S.; Day, T. J. F.; Honig, B.; Shaw, D. E.; Friesner, R. A. A hierarchical approach to all-atom protein loop prediction. *Proteins: Structure, Function, and Bioinformatics* **2004,** *55,* 351–367, eprint: https://onlinelibrary.wiley.com/doi/pdf/10.1002/prot.10613.
