# Supplementary figures and images for: Covalent docking and molecular dynamics simulations reveal the specificity-shifting mutations Ala237Arg and Ala237Lys in TEM beta-lactamase
Source: PLoS Comput Biol. 2022 Jun 27;18(6):e1009944. doi: 10.1371/journal.pcbi.1009944 (PMC9269908; doi:10.1371/journal.pcbi.1009944)

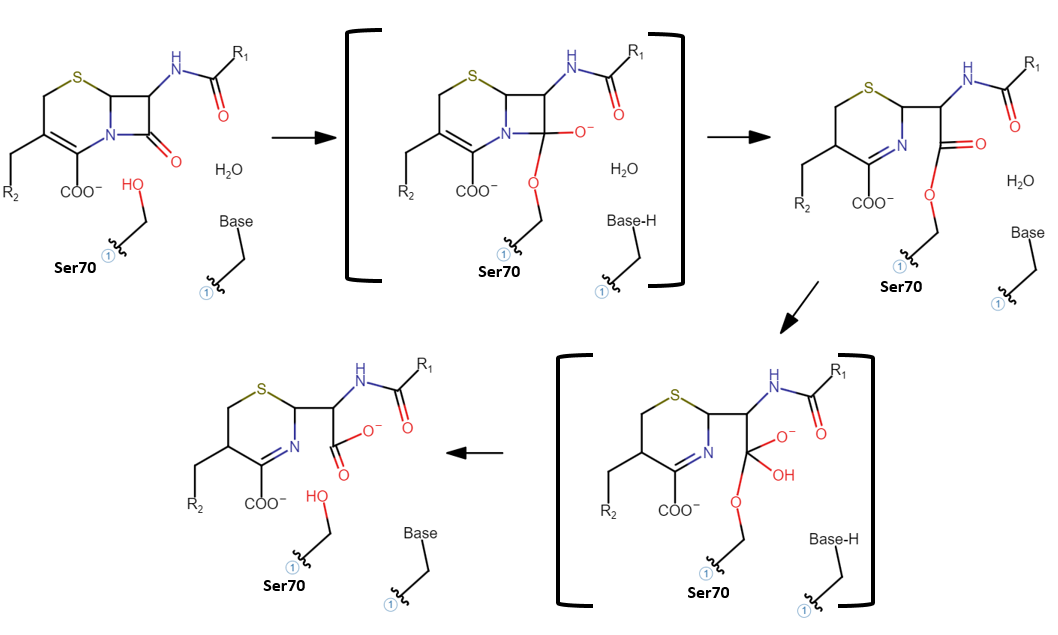

Supplement: S1 Fig — (PNG) [file pcbi.1009944.s002.png]

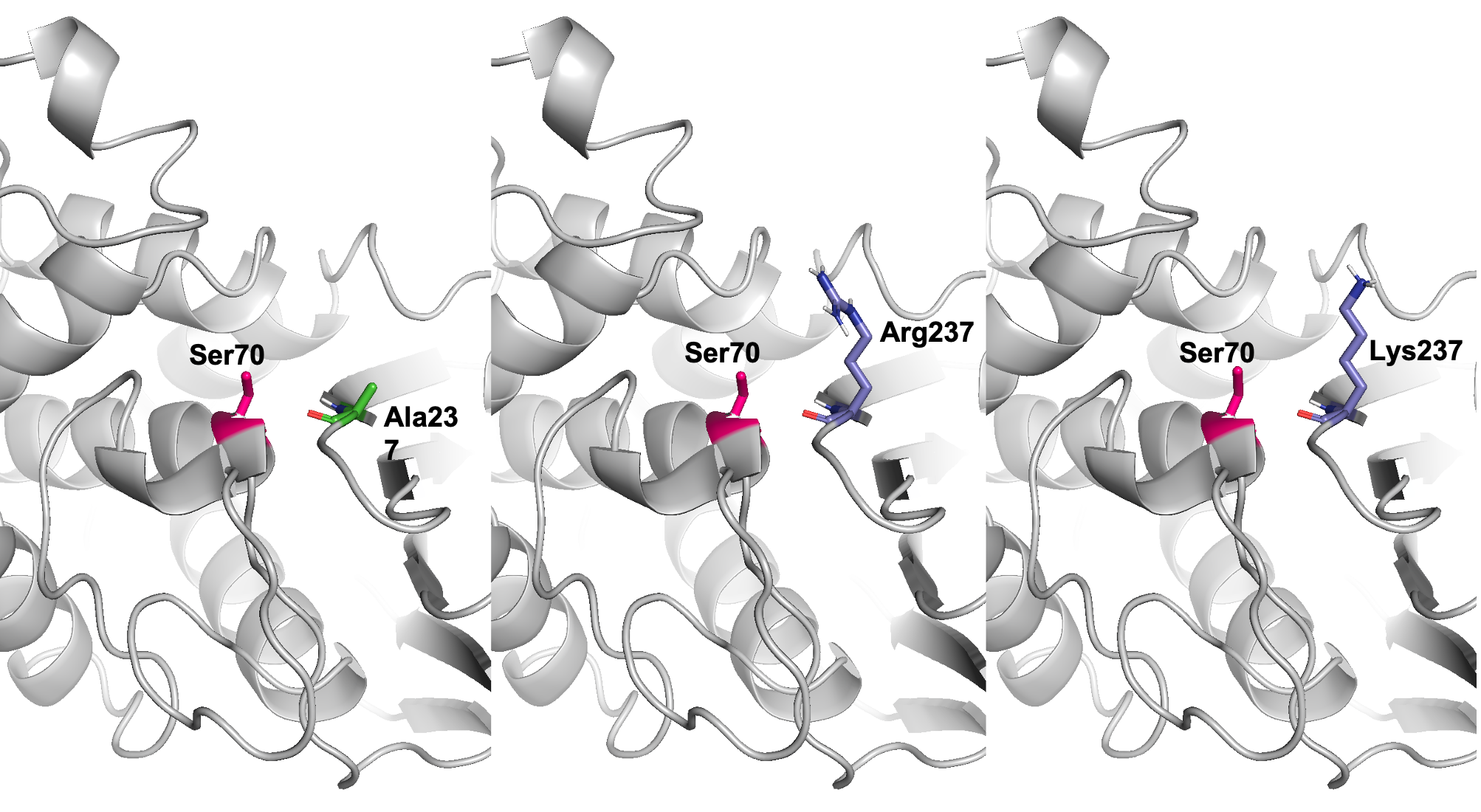

Supplement: S2 Fig — Serine 70, the catalytic residue, is represented in fuchsia. (PNG) [file pcbi.1009944.s003.png]

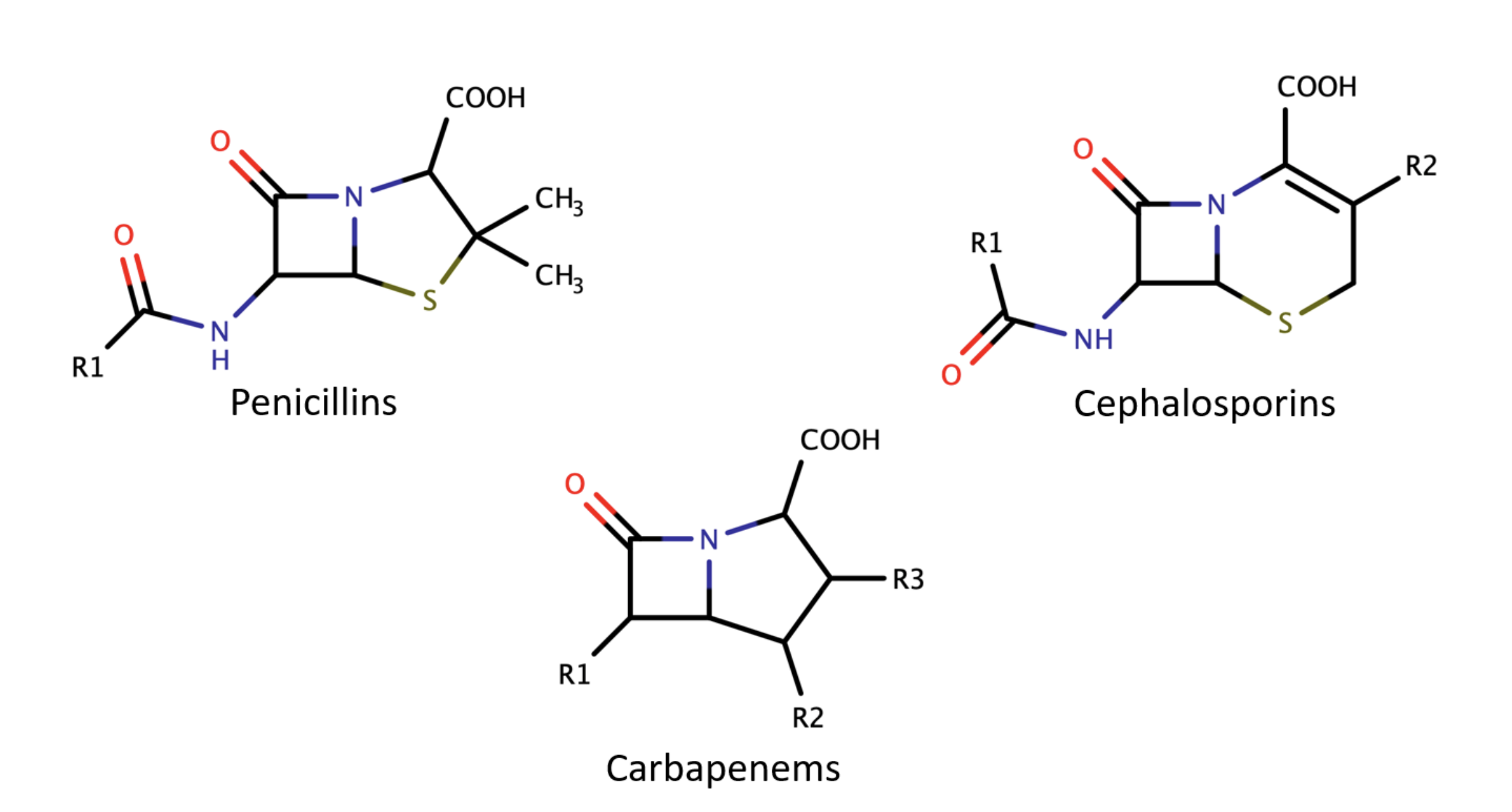

Supplement: S3 Fig — (PNG) [file pcbi.1009944.s004.png]
