# Supplementary material for: Covalent docking and molecular dynamics simulations reveal the specificity-shifting mutations Ala237Arg and Ala237Lys in TEM beta-lactamase
Source: PLoS Comput Biol. 2022 Jun 27;18(6):e1009944. doi: 10.1371/journal.pcbi.1009944 (PMC9269908; doi:10.1371/journal.pcbi.1009944)
Supplement: S1 Table — (PDF) [file pcbi.1009944.s005.pdf]

Table S1: Sequences for the mutagenesis primers used to generate the 20 TEM Ala237X constructs.

Mutation	Mut_F_Primer
Ala237Arg	ACCCACGCTCACCGCGTCCAGATTTATCAGCAATAAAC
Ala237Asn	GACCCACGCTCACCGTTTCCAGATTTATCAGCAATAAACCA
Ala237Asp	GACCCACGCTCACCGTCTCCAGATTTATCAG
Ala237Cys	GACCCACGCTCACCGCATCCAGATTTATCAGCAATAAACCA
Ala237Gln	GAGACCCACGCTCACCCTGTCCAGATTTATCAGCAATAAACCA
Ala237Glu	GAGACCCACGCTCACCCTCTCCAGATTTATCAGC
Ala237Gly	GACCCACGCTCACCGCCTCCAGATTTATCAG
Ala237His	GACCCACGCTCACCGTGTCCAGATTTATCAGCAATAAACCA
Ala237Ile	GACCCACGCTCACCGATTCCAGATTTATCAGCAATAAACCA
Ala237Leu	CGAGACCCACGCTCACCTAGTCCAGATTTATCAGCAATAAACCAGC
Ala237Lys	CGAGACCCACGCTCACCCTTTCCAGATTTATCAGCAATAAACCAGC
Ala237Met	CGAGACCCACGCTCACCCATTCCAGATTTATCAGCAATAAACCAGC
Ala237Phe	GACCCACGCTCACCGAATCCAGATTTATCAGCAATAAACCA
Ala237Pro	CCACGCTCACCGGGTCCAGATTTATCAGCAATAAAC
Ala237Ser	GACCCACGCTCACCGCTTCCAGATTTATCAGCAATAAACCA
Ala237Thr	CCACGCTCACCGGTTCCAGATTTATCAGCAATAAAC
Ala237Trp	GAGACCCACGCTCACCCCATCCAGATTTATCAGCAATAAACCA
Ala237Tyr	CGAGACCCACGCTCACCATATCCAGATTTATCAGCAATAAACCAGC
Ala237Val	GACCCACGCTCACCGACTCCAGATTTATCAG

Mutation	Mut_R_Primer
Ala237Arg	GTTTATTGCTGATAAATCTGGACGCGGTGAGCGTGGGT
Ala237Asn	TGGTTTATTGCTGATAAATCTGGAAACGGTGAGCGTGGGTC
Ala237Asp	CTGATAAATCTGGAGACGGTGAGCGTGGGTC
Ala237Cys	TGGTTTATTGCTGATAAATCTGGATGCGGTGAGCGTGGGTC
Ala237Gln	TGGTTTATTGCTGATAAATCTGGACAGGGTGAGCGTGGGTCTC
Ala237Glu	GCTGATAAATCTGGAGAGGGTGAGCGTGGGTCTC
Ala237Gly	CTGATAAATCTGGAGGCGGTGAGCGTGGGTC
Ala237His	TGGTTTATTGCTGATAAATCTGGACACGGTGAGCGTGGGTC
Ala237Ile	TGGTTTATTGCTGATAAATCTGGAATCGGTGAGCGTGGGTC
Ala237Leu	GCTGGTTTATTGCTGATAAATCTGGACTAGGTGAGCGTGGGTCTCG
Ala237Lys	GCTGGTTTATTGCTGATAAATCTGGAAAGGGTGAGCGTGGGTCTCG
Ala237Met	GCTGGTTTATTGCTGATAAATCTGGAATGGGTGAGCGTGGGTCTCG
Ala237Phe	TGGTTTATTGCTGATAAATCTGGATTCGGTGAGCGTGGGTC
Ala237Pro	GTTTATTGCTGATAAATCTGGACCCGGTGAGCGTGG
Ala237Ser	TGGTTTATTGCTGATAAATCTGGAAGCGGTGAGCGTGGGTC
Ala237Thr	GTTTATTGCTGATAAATCTGGAACCGGTGAGCGTGG
Ala237Trp	TGGTTTATTGCTGATAAATCTGGATGGGGTGAGCGTGGGTCTC
Ala237Tyr	GCTGGTTTATTGCTGATAAATCTGGATATGGTGAGCGTGGGTCTCG
Ala237Val	CTGATAAATCTGGAGTCGGTGAGCGTGGGTC
