# Supplementary material for: Covalent docking and molecular dynamics simulations reveal the specificity-shifting mutations Ala237Arg and Ala237Lys in TEM beta-lactamase
Source: PLoS Comput Biol. 2022 Jun 27;18(6):e1009944. doi: 10.1371/journal.pcbi.1009944 (PMC9269908; doi:10.1371/journal.pcbi.1009944)
Supplement: S2 Table — (PDF) [file pcbi.1009944.s006.pdf]

Table S2: List of all drugs used in the CovDock predictions

Compound	Class	PubChem CID
Amoxicillin	Aminopenicillin	33613
Ampicillin	Aminopenicillin	6249
Bacampicillin	Aminopenicillin	441397
Epicillin	Aminopenicillin	71392
Hetacillin	Aminopenicillin	443387
Metampicillin	Aminopenicillin	6713928
Pivampicillin	Aminopenicillin	33478
Talampicillin	Aminopenicillin	71447
Biapenem	Carbapenems	71339
Doripenem	Carbapenems	73303
Ertapenem	Carbapenems	150610
Imipenem	Carbapenems	104838
Meropenem	Carbapenems	441130
Panipenem	Carbapenems	72015
Cefacetrile	Cephalosporins1	91562
Cefadroxil	Cephalosporins1	47965
Cefalexin	Cephalosporins1	27447
Cefaloglycin	Cephalosporins1	19150
Cefalonium	Cephalosporins1	21743
Cefaloridine	Cephalosporins1	5773
Cefalotin	Cephalosporins1	6024
Cefapirin	Cephalosporins1	30699
Cefatrizine	Cephalosporins1	6410758
Cefazaflur	Cephalosporins1	40240

Cefazedone	Cephalosporins1	71736
Cefazolin	Cephalosporins1	33255
Cefradine	Cephalosporins1	38103
Cefroxadine	Cephalosporins1	5284529
Ceftezole	Cephalosporins1	65755
Cefaclor	Cephalosporins2	51039
Cefamandole	Cephalosporins2	456255
Cefbuperazone	Cephalosporins2	127527
Cefminox	Cephalosporins2	71141
Cefonicid	Cephalosporins2	43594
Ceforanide	Cephalosporins2	43507
Cefotetan	Cephalosporins2	53025
Cefoxitin	Cephalosporins2	441199
Cefprozil	Cephalosporins2	5281006
Cefuroxime	Cephalosporins2	5479529
Cefuroxime Axetil	Cephalosporins2	6321416
Cefuzonam	Cephalosporins2	6336505
Loracarbef	Cephalosporins2	5284585
Cefcapene	Cephalosporins3	6436055
Cefdaloxime	Cephalosporins3	9571072
Cefdinir	Cephalosporins3	6915944
Cefetamet	Cephalosporins3	5487888
Cefixime	Cephalosporins3	5362065
Cefmenoxime	Cephalosporins3	9570757
Cefodizime	Cephalosporins3	5361871
Cefoperazone	Cephalosporins3	44187
Cefotaxime	Cephalosporins3	5742673

Cefotiam	Cephalosporins3	43708
Cefpimizole	Cephalosporins3	68597
Cefsulodin	Cephalosporins3	656575
Ceftazidime	Cephalosporins3	5481173
Cefteram	Cephalosporins3	6537431
Ceftibuten	Cephalosporins3	5282242
Ceftiolene	Cephalosporins3	6537430
Flomoxef	Cephalosporins3	65864
Latamoxef	Cephalosporins3	47499
Cefepime	Cephalosporins4	5479537
Cefozopran	Cephalosporins4	9571080
Cefpirome	Cephalosporins4	5479539
Cefquinome	Cephalosporins4	5464355
Ceftaroline Fosamil	Cephalosporins5	9852981
Ceftobiprole	Cephalosporins5	135413542
Ceftolozane	Cephalosporins5	53234134
Aztreonam	Monobactams	5742832
Carumonam	Monobactams	6540466
NocardicinA	Monobactams	6419429
Tigemonam	Monobactams	9576769
Azidocillin	Penicillins1	15574941
Benzathine Benzylpenicillin	Penicillins1	15232
Benzylpenicillin	Penicillins1	5904
Clometocillin	Penicillins1	71807
Penamecillin	Penicillins1	10250769
Pheneticillin	Penicillins1	272833
Phenoxymethyl Penicillin	Penicillins1	6869

Procaine Benzylpenicillin	Penicillins1	5903
Propicillin	Penicillins1	92879
Cloxacillin	Penicillins2	6098
Methicillin	Penicillins2	6087
Nafcillin	Penicillins2	8982
Oxacillin	Penicillins2	6196
Azlocillin	Penicillins4	6479523
Carbenicillin	Penicillins4	20824
Carindacillin	Penicillins4	93184
Mezlocillin	Penicillins4	656511
Piperacillin	Penicillins4	43672
Temocillin	Penicillins4	171758
Ticarcillin	Penicillins4	36921
