# Supplementary material for: Covalent docking and molecular dynamics simulations reveal the specificity-shifting mutations Ala237Arg and Ala237Lys in TEM beta-lactamase
Source: PLoS Comput Biol. 2022 Jun 27;18(6):e1009944. doi: 10.1371/journal.pcbi.1009944 (PMC9269908; doi:10.1371/journal.pcbi.1009944)
Supplement: S3 Table — (PDF) [file pcbi.1009944.s007.pdf]

Table S3: CovDock scores (in kcal/mol) for the three compounds investigated in detail in this study against TEM-1 and TEM Ala237Arg/Lys.

CovDock Scores (kcal/mol)	TEM-1	TEM-Ala237Arg	TEM-Ala237Lys
**Ampicillin**	-6.308	-3.874	-4.479
**Cefixime**	-3.559	-5.403	-5.52
**Ceftibuten**	-4.99	-6.465	-6.681
**Carumonam**	-3.256	-4.595	-3.817
