# Supplementary material for: Covalent docking and molecular dynamics simulations reveal the specificity-shifting mutations Ala237Arg and Ala237Lys in TEM beta-lactamase
Source: PLoS Comput Biol. 2022 Jun 27;18(6):e1009944. doi: 10.1371/journal.pcbi.1009944 (PMC9269908; doi:10.1371/journal.pcbi.1009944)
Supplement: S4 Table — Mutants where no docking score was calculated due to extremely unfavorable poses had their scores set as” NAN”. (PDF) [file pcbi.1009944.s008.pdf]

**Table S4: Relative docking scores and fitness values for all tested mutants upon treatment with wither ampicillin or cefixime.** Mutants where no docking score was calculated due to extremely unfavorable poses had their scores set as ”NAN”.

| Mutation | Rel. Docking Score | Rel. Fitness | Drug |
|---|---|---|---|
| A237C | 1.031547 | 0.5 | Ampicillin |
| A237D | 0.771084 | 0.25 | Ampicillin |
| A237E | 0.950698 | 0.25 | Ampicillin |
| A237F | NAN | 0.125 | Ampicillin |
| A237G | 0.934686 | 1 | Ampicillin |
| A237H | NAN | 0.125 | Ampicillin |
| A237I | 0.907261 | 0.25 | Ampicillin |
| A237K | 0.710051 | 0.125 | Ampicillin |
| A237L | 0.980659 | 1 | Ampicillin |
| A237M | 0.882091 | 0.5 | Ampicillin |
| A237N | 0.954661 | 0.5 | Ampicillin |
| A237P | 0.462904 | 0.0625 | Ampicillin |
| A237Q | 0.911699 | 0.25 | Ampicillin |
| A237R | 0.614141 | 0.125 | Ampicillin |
| A237S | 1.004756 | 1 | Ampicillin |
| A237T | 0.987952 | 2 | Ampicillin |
| A237V | 1.012841 | 0.5 | Ampicillin |
| A237W | -0.05696 | 0.5 | Ampicillin |
| A237Y | NAN | 0.25 | Ampicillin |
| A237C | 1.198388 | 1 | Cefixime |
| A237D | 0.913865 | 1 | Cefixime |
| A237E | 1.078911 | 1 | Cefixime |
| A237F | 0.894971 | 1 | Cefixime |
| A237G | 1.044735 | 1 | Cefixime |
| A237H | 1.087802 | 1 | Cefixime |
| A237I | 1.155599 | 1 | Cefixime |
| A237K | 1.533759 | 2 | Cefixime |
| A237L | 1.298138 | 1 | Cefixime |
| A237M | 1.01923 | 1 | Cefixime |
| A237N | 1.36788 | 1 | Cefixime |
| A237P | -0.05696 | 1 | Cefixime |
| A237Q | 1.351487 | 1 | Cefixime |
| A237R | 1.50125 | 4 | Cefixime |
| A237S | 1.103362 | 1 | Cefixime |
| A237T | 1.234787 | 2 | Cefixime |
| A237V | 1.562934 | 1 | Cefixime |
| A237W | NAN | 1 | Cefixime |
| A237Y | 1.061684 | 1 | Cefixime |
