# Supplementary material for: Covalent docking and molecular dynamics simulations reveal the specificity-shifting mutations Ala237Arg and Ala237Lys in TEM beta-lactamase
Source: PLoS Comput Biol. 2022 Jun 27;18(6):e1009944. doi: 10.1371/journal.pcbi.1009944 (PMC9269908; doi:10.1371/journal.pcbi.1009944)
Supplement: Attachment — Submitted filename: gms_beta_lactamase_plos_compbio_letter_to_reviewers.pdf [file pcbi.1009944.s010.pdf]

BROWN
Department of Chemistry

Brown University
Department of Chemistry
324 Brook Street, Box H
Providence, Rhode Island 02912, USA
Tel: (603) 661-2160
E-mail: Brenda_Rubenstein@brown.edu

Brenda Rubenstein
https://www.brown.edu/research/labs/rubenstein/home
Associate Professor of Chemistry

May 10, 2022

Dear Editor,

First of all, we thank the reviewers for their time and effort carefully reviewing and analyzing our manuscript, particularly during this challenging time. We appreciate their many useful suggestions, which will improve the quality of our manuscript. In what follows, we respond to the reviewers' comments one-by-one in blue.

Reviewer #1:

This manuscript explores how mutations give rise to new forms of beta-lactamase-mediated antibiotic resistance. It is a nice combination of computational methods with experimental tests of new predictions. The approach and insights could both help accelerate the development of new antibiotics to keep pace with the evolution of resistance.

We thank the reviewer for recognizing and highlighting the significance of this work. We agree that our insights can potentially accelerate the development of new antibiotics.

- Briefly explain CovDock at the beginning of the results, or in the introduction. In particular, how is the covalent linkage enforced? Is there receptor flexibility? Is the ligand flexibility simulated on the fly, or pre-enumerated?

Thank you for your feedback. We agree that these details are crucial for understanding the significance of our results and for improving reproducibility. We have added extensive documentation regarding CovDock's methodology to the main text of the manuscript as suggested (lines 166-171), quoted below:

CovDock is a covalent docking algorithm developed by Schrödinger, Inc., that uses the Glide docking engine coupled to the Prime molecular modeling software to covalently dock compounds. As summarized in Figure 1, the CovDock workflow starts by mutating the reactive residue (in this case, Serine 70) to alanine, then enumerates ligand conformations, docks the ligand to a flexible receptor with positional restraints (to ensure the formation of the covalent bond), restores the reactive residue to a rotamer predicted to be favorable, and forms the covalent bond. Finally, the energy of the binding site is minimized and docking scores for the covalent poses are calculated with Glide.

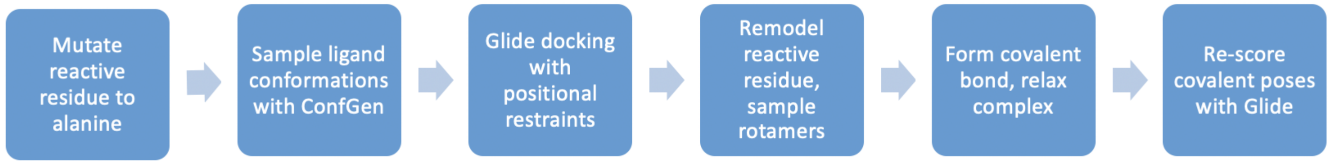

Figure 1: Schrodinger's CovDock workflow for covalent docking, adapted from Schrodinger's documentation

- From figure 1, it looks like Val, Leu, Gln, and Ile similar/more enhanced binding to cephalosporins. Is that not the case? If it is, what's going on there? And why the focus on Lys/Arg?

The arginine and lysine mutations result in the largest binding changes, which are corroborated by the IC-50 experiments with cefixime and ampicillin. Although we are definitely interested in the potential effects of other mutations, the fact that the experiment did not corroborate our predictions for these mutations led us to avoid speculating about their effects in the discussion.

- Are Ala237Lys/Arg mutations ever seen in the clinic? If not, why to the authors suppose that's the case?

Ala237Lys is of significant importance in the clinic as part of the TEM-24 compound mutant (Leu102Lys, Leu162Ser, Ser235Thr, Ala237Lys)[1] , but Ala237Arg or Ala237Lys by themselves have not been reported. We believe that this happens due to the fact that, while these mutations greatly improve the binding and catalysis of certain cephalosporins, they drastically reduce the binding of most beta-lactam antibiotics due to the unfavorable steric clashes presented by the bulky and charged chemical shifts. The ultimate fitness outcome is likely negative for bacteria expressing TEM Ala237Lys/Arg upon most commonly-used antibiotic treatments.

1. Galdbart JO, Lémann F, Ainouz D, Féron P, Lambert-Zechovsky N, Branger C. TEM-24 extended-spectrum beta-lactamase-producing Enterobacter aerogenes: long-term clonal dissemination in French hospitals. Clin Microbiol Infect. 2000 Jun;6(6):316-23. doi: 10.1046/j.1469-0691.2000.00092.x. PMID: 11168140.

- Need error bars for all plots and results reported, so that the reader can judge their statistical significance.

Thank you for the suggestion. We have reported standard deviations for all of the measurements calculated in replicates. Figures 6, 7, and 8 have been updated in the main text to reflect this change.

- The correlation between the docking score and MIC is so low that I bet it is within error of zero. Either way, it may not be worth reporting, since the main conclusions are based on clear outliers (which are probably statistically significant), not a trend (which is probably absent).

We absolutely agree that the correlation between prediction and experiment is extremely weak and that the interesting findings pertain to the outliers.

We made the decision to include this figure because it transparently shows the inherent limitations of comparing binding predictions to fitness experiments, as well as the large approximations of docking

algorithms (even those as sophisticated as CovDock). We have modified the discussion to further emphasize this (line 277, quoted below).

Although the overall correlation is very weak, not allowing for broad hypotheses spanning all possible mutants, we identified very interesting patterns for a few substitutions.

- The results would be much stronger if the authors did the MIC measurements for the two new compounds, Cet and Car.

We agree with this assessment and we are considering further investigating the effects of the mutations in the binding of cefotaxime and carumonam in future studies.

- The conclusions make the very interesting claim that Ala237 is highly conserved but that mutations can still increase fitness. Please show data to support the claim that the residue is conserved, compared to elsewhere in the protein or maybe even relative to other active site residues?

Thank you for the suggestion. We have included a phylogenetic analysis that shows the mostly conserved status of Ala237 in TEM beta-lactamase in the main text (lines 225-227; describing the results, and lines 432-437; describing the methods), quoted below:

Importantly, Alanine 237 is conserved amongst species that express TEM, with a PRCP conservation score (10.1093/bioinformatics/btm270) of 0.6 calculated from the 50 entries most similar to E. Coli's TEM-1 in the UniProt database (UniProt ID P62593) (10.1093/nar/gkaa1100).

Conservation Analysis of Alanine 237

To measure the conservation level of Ala237 across different species expressing TEM beta-lactamase, we queried the UniProt database (10.1093/nar/gkaa1100) for proteins with sequences similar to E. Coli's TEM-1 sequence (UniProt ID P62593). We then generated a multiple sequence alignment of the 50 most similar entries using the CLUSTAL W algorithm (Thompson1994), and used the Protein Residue Conservation Prediction webserver (10.1093/bioinformatics/btm270) to calculate the conservation score of Alanine 237 based on the previously generated alignment.

Reviewer #2:

Monteiro da Silva and co-authors present a study on the effects of Ala237 mutations in TEM beta-lactamases. After performing covalent docking to screen 19 residue mutations at this position against a panel of 91 beta-lactam antibiotics, the authors further analyze the effects of A237R and A237K mutations with molecular dynamics simulations and Minimum Inhibitory Concentration (MIC) assays. Overall, this study provides convincing evidence of the opposite effects of these mutations against different classes of beta-lactam antibiotics and their underlying molecular mechanism. The manuscript is concise and clearly written, the work appears technically sound, and the discussion of the results is generally well balanced and measured with respect to the reliability of the different approaches and the overall evidence from current and past data.

We thank the reviewer for their supportive comments.

Because of this, my opinion is that the manuscript is suitable for publication, although after a few minor revisions. My two main suggestions concern uncertainty estimation and coverage of past literature:

1) All results (figures 5, 6, and 7) concerning molecular dynamics simulations should report some measure of uncertainty/variation, such as mean and standard error. The qualitative difference observed seem to indicate that the different would also be statistically significant, but given the stochasticity of MD simulations, is importance to quantify the uncertainty associated with the observables studied. As such, Figure 5 should show the standard error of the mean or some confidence interval. Figures 6 and 7 should also show mean +/- uncertainty across the 10 repeats, rather than being taken from a single trajectory.

Thank you for the feedback. We definitely agree that it is important to measure the significance of our results and thus have calculated confidence intervals for the results of the binding pose metadynamics simulations and updated Figures 6 through 8 (previously 5 to 8) to reflect this information. Despite the high variance - which is to be expected from accelerated MD - the significance of our results is preserved as there are stark contrasts between the stability of cefixime or ampicillin bound to either TEM-1 or TEM-Ala237Arg, even when including data for the 10 replicates. Also of note, in the process of regenerating these figures, we realized that the previously shown CV RMSD data didn’t actually include data from all of the 10 ns simulations. The new figure (shown directly below) includes information from all ten replicates.

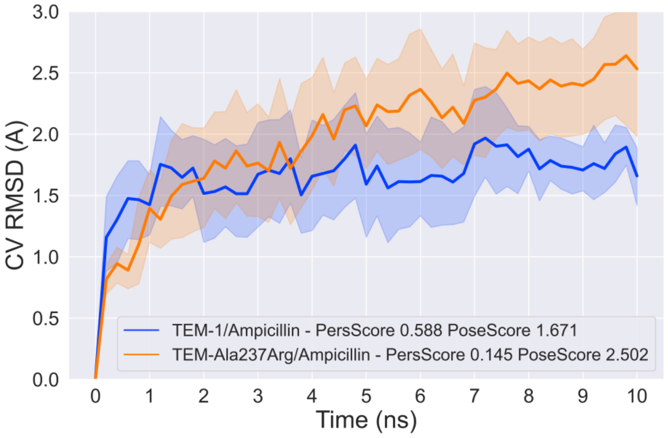

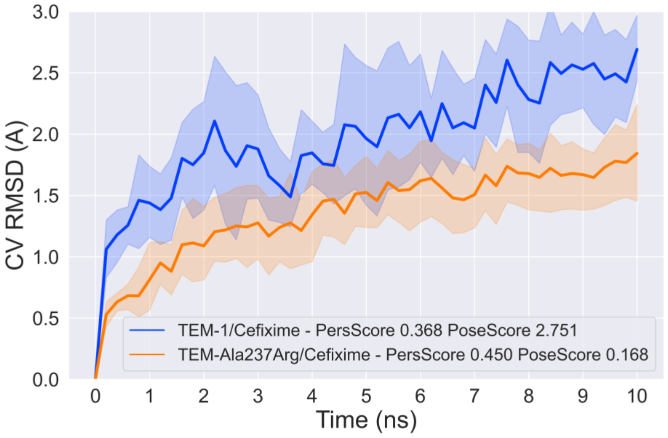

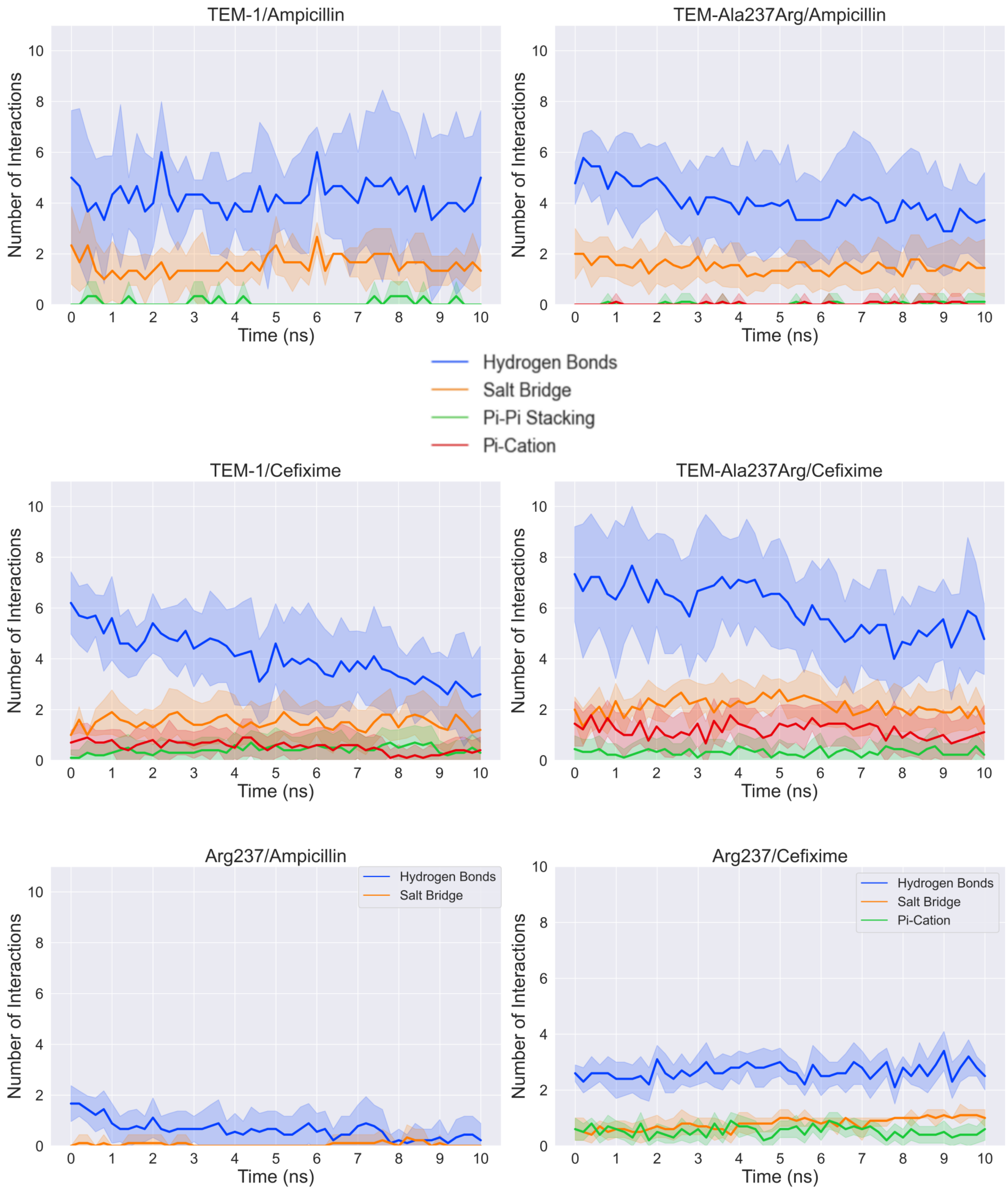

TEM-1/Ampicillin
TEM-Ala237Arg/Ampicillin
Hydrogen Bonds
Salt Bridge
Pi-Pi Stacking
Pi-Cation
TEM-1/Cefixime
TEM-Ala237Arg/Cefixime
Arg237/Ampicillin
Arg237/Cefixime
Number of Interactions
Time (ns)

2) There have been several recent works on predicting drug resistance computationally via molecular modeling approaches. While the authors do cite some reviews in the introduction, I think it is appropriate to cite the relevant primary literature too in a more comprehensive appraisal of the field. This is a list of the studies I am aware of, but there are likely others:

Hauser et al. "Predicting resistance of clinical Abl mutations to targeted kinase inhibitors using alchemical free-energy calculations" Commun. Biol. 2018

Fowler et al. "Robust Prediction of Resistance to Trimethoprim in Staphylococcus aureus" Cell Chem. Biol. 2018

Aldeghi et al. "Accurate Estimation of Ligand Binding Affinity Changes upon Protein Mutation" ACS Cent. Sci. 2018

Aldeghi et al. "Predicting Kinase Inhibitor Resistance: Physics-Based and Data-Driven Approaches", ACS Cent. Sci. 2019

Frey et al. "Predicting resistance mutations using protein design algorithms", PNAS 2010

Brankin & Fowler "Predicting antibiotic resistance in complex protein targets using alchemical free energy methods", ChemRxiv 2021

Sun et al "PremPLI: a machine learning model for predicting the effects of missense mutations on protein-ligand interactions" Commun. Biol. 2021

Yang et al. "SPLDExtraTrees: robust machine learning approach for predicting kinase inhibitor resistance" Brief. Bioinform. 2022

Thank you for the feedback and for compiling this list of relevant references. This is extremely helpful. We have cited the suggested studies in the text, in the fourth paragraph of the Introduction where current and past methods for predicting binding and fitness landscapes are discussed.

Other minor comments and suggestions:

- The validation of some of the docking results with more quantitative computational approaches, like free energy perturbation, could be a valuable and complementary addition to the study. I do not think it is strictly necessary for this work, however. This could be a consideration for future work.

Thank you for the suggestion. We absolutely agree that FEP would be very useful to validate our proposed mechanisms and are contemplating using it in the context of future studies exploring a narrower range of mutations with unexpected and interesting effects on binding. For this study in specific, we relied more on high-throughput prediction methods (FEP is substantially more expensive than the techniques used here) such as docking and short MD simulations because of the throughput needed for our studies and due to the exploratory nature of our hypotheses.

- It looks like Figure S1 might be missing from the SI, as it does not match the description at page 5. Fig. S1 is about "Schrodinger's CovDock workflow" but it is described in the text as "the side-chain oxygen of TEM's Ser70 residue binds to the /3-lactam's carbonyl via nucleophilic attack".

Thanks for letting us know about this issue. We have updated the manuscript with the appropriate corrections. Per the reviewer's feedback, we moved Figure S1 (which was missing) to the main text as Figure 1. Figure S1 is now correctly labeled and included in the supporting information.

- There may be the opportunity to better highlight in Figures 1-3 which values correspond to "better vs worse" scores/fitness. E.g., in Figures 1 and 2, having the color bar be white when the relative score is equal to 1 (red when less than one, and blue when above one) would allow more immediately identify values close or slightly above/below 1; in Figure 3, perhaps one could label the relative fitness of 1 being the WT, with values above 1 meaning resistant mutations (worse drug efficacy), and below 1 sensitizing ones (better drug efficacy).

Thank you for the very detailed feedback. We have made changes to Figures 1 and 2 to better reflect the predicted changes in binding affinity (the color bar is now centered at 1 as suggested). We have also added dotted lines for both axes in Figure 3 indicating the "wild-type" value for either measurement, which we believe should facilitate visualization by separating the plot into quadrants. Feel free to suggest further changes if this is insufficient.

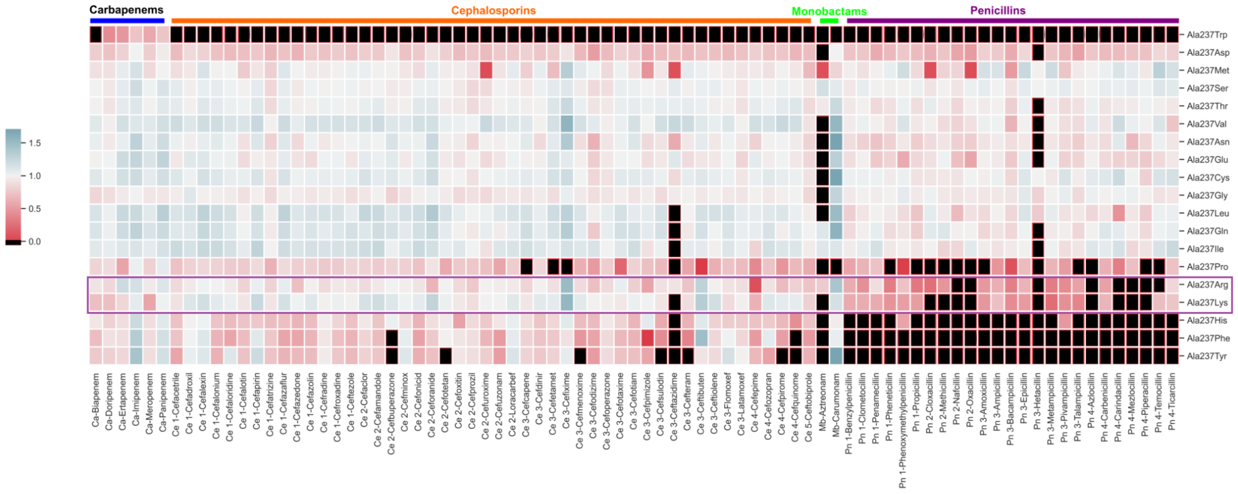

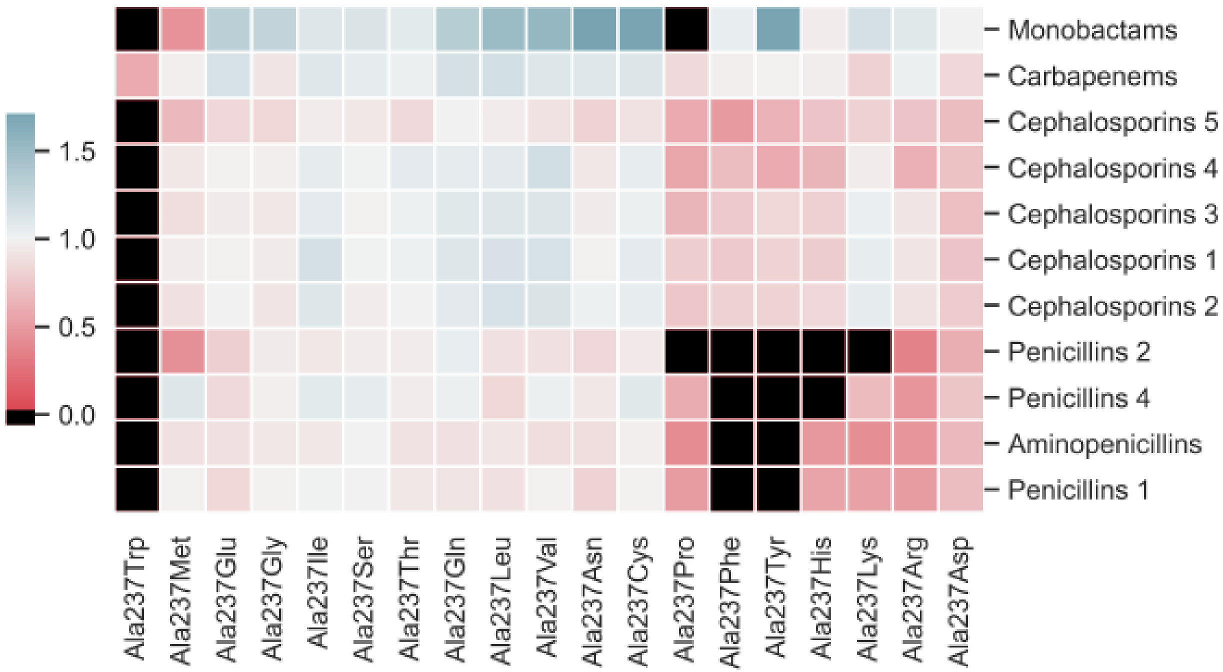

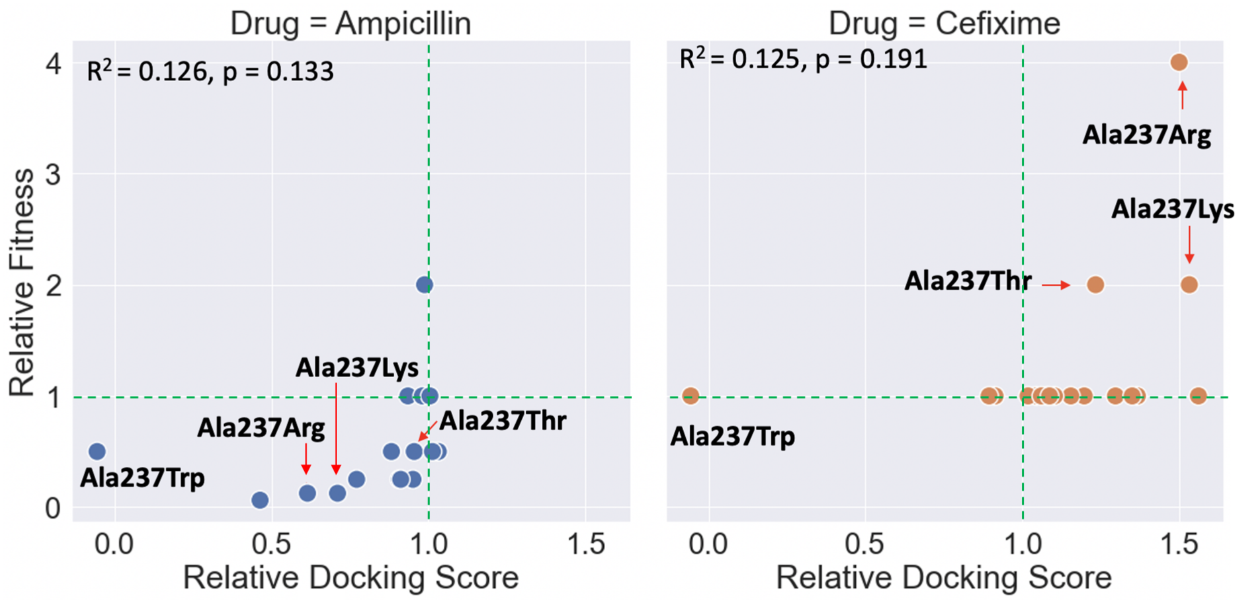

- In Figure 5, the PersScore and PoseScore are reported, but it is not clear what these are if one is not already familiar with them. It would be beneficial to add a brief explanation of these in the text or the figure caption directly.

&

- Somewhat related to the point above, from the Methods it is not clear how the PersScore and PoseScore are calculated. It would be good to add more details such that the reader will know what these scores are capturing more exactly.

Thank you for the suggestion. We have added a concise description of what PersScore and PoseScore mean and how they are calculated to the main text of the manuscript, in the caption of Figure 6 (previously called Figure 5), and in the Methods Section (lines 490-496), quoted below.

Figure 6. Binding pose metadynamics results for complexes with ampicillin (left) and cefixime (right). In addition to the ligand RMSD, for each set of simulations, we also report a measurement of the stability of the network of hydrogen bonds in the pre-simulation pose, called the PersScore, and the ligand RMSD for the final frame of the simulation in regards to the initial pose, called the PoseScore. All data shown are averaged over ten replicates. The shading denotes the standard deviation of the RMSD values for each system.

The PersScore term is calculated by measuring the fraction of frames in the last two nanoseconds of the simulations that have the same number of hydrogen bonds between the ligand and the receptor as the input structure, averaged over all of the replicates. Meanwhile, the PoseScore term is calculated by measuring the ligand's RMSD for the last frame of the simulation relative to its initial pose, also averaged across all replicates.

We thank the reviewers again for their thoughtful comments. We hope that we have addressed your primary concerns.

Yours truly,

Associate Professor of Chemistry
Brown University

Ph.D. Student, Molecular and Celular Biology
Brown University
